# Enhancement of DNaseI Salt Tolerance by Mimicking the Domain Structure of DNase from an Extremely Halotolerant Bacterium *Thioalkalivibrio sp. K90mix*

**DOI:** 10.1371/journal.pone.0150404

**Published:** 2016-03-03

**Authors:** Gediminas Alzbutas, Milda Kaniusaite, Arunas Lagunavicius

**Affiliations:** 1 VU Institute of Biotechnology, V.A. Graiciuno 8, LT-02241 Vilnius, Lithuania; 2 Thermo Fisher Scientific, V.A. Graiciuno 8, LT-02241 Vilnius, Lithuania; Institute of Molecular Genetics IMG-CNR, ITALY

## Abstract

In our previous work we showed that DNaseI-like protein from an extremely halotolerant bacterium *Thioalkalivibrio sp. K90mix* retained its activity at salt concentrations as high as 4 M NaCl and the key factor allowing this was the C-terminal DNA-binding domain, which comprised two HhH (helix-hairpin-helix) motifs. The further investigations revealed that this domain originated from proteins related to bacterial competence ComEA/ComE proteins. It is likely that in the course of evolution the DNA-binding domain from these proteins was fused to a metallo-*β*-lactamase superfamily domain. Very likely such domain organization having proteins subsequently “donated” the DNA-binding domain to bacterial DNases. In this study we have mimicked this evolutionary step by fusing bovine DNaseI and DNA-binding domains. We have created two fusions: one harboring the DNA-binding domain of DNaseI-like protein from *Thioalkalivibrio sp. K90mix* and the second one harboring the DNA-binding domain of bacterial competence protein ComEA from *Bacillus subtilis*. Both domains enhanced salt tolerance of DNaseI, albeit to different extent. Molecular modeling revealed the essential differences between their interaction with DNA shedding some light on the differences in salt tolerance. In this study we have enhanced salt tolerance of bovine DNaseI; thus, we successfully mimicked the Nature’s evolutionary engineering that created the extremely halotolerant bacterial DNase. We have demonstrated that the newly engineered DNaseI variants can be successfully used in applications where activity of the wild type bovine DNaseI is impeded by buffers used.

## Introduction

In molecular biology research deoxyribonuclease I (DNaseI) is used in several applications, such as removal of genomic DNA from cell lysates, removal of plasmid from in vitro transcribed RNA, nick translation, DNaseI footprinting, RNA purification and quantification. In many cases buffers that are used in such applications contain substantial amount of salt. A well known drawback of the commonly used bovine DNaseI is its poor tolerance to ionic strength. For example, in order to efficiently remove residual genomic DNA during RNA purification high DNaseI concentrations are required or, alternatively, an explicit DNaseI treatment of isolated RNA sample must be done. However, the additional step comes with substantial increase in hands-on time.

It is known that an increase in ionic strength usually hinders DNA—protein interactions. One way of coping with this is to introduce additional positive residues onto DNA-binding surface onto the enzyme surface that interacts with DNA. In the past this approach yielded salt resistant variants of eukaryotic DNaseI [1, 2].

An alternative approach to enhance DNA-binding has been used with several other enzymes in order to alter their properties. In this case an enzyme is fused with a non-specific DNA-binding domain. In such a way affinity for DNA and, consequently, various properties were improved for several DNA polymerases: Phage phi 29 DNA polymerase [3], Taq and Pfu DNA polymerases [4, 5]. However DNA-binding mode by DNA polymerases is fundamentally different from that of DNaseI: DNA polymerases sequentially add nucleotides to extend the 3′ end of an oligonucleotide, while DNaseI cleaves phosphodiester bonds in a non-sequential manner. Therefore, the knowledge that was derived from the successful generation of useful chimeric DNA polymerases cannot be directly applied for construction of chimeric DNaseI proteins having enhanced DNA-binding properties. Moreover, a large number of DNA-binding domains and proteins are known from across all domains of life and viruses; however, the current state of the art provides little guidance to assist a researcher to choose a fusion partner for DNaseI.

Nevertheless, in this article we report on successful efforts to create fusion comprising DNaseI and DNA-binding domain. In such a way enhanced DNA-binding by DNaseI enabled retention of hydrolytic activity at extremely high ionic strength. In this way we mimicked domain architecture of an extremely salt tolerant DNase [6] (DNaseTA) from a salt tolerant bacterium *Thioalkalivibrio sp. K90mix*. DNaseTA is comprised of two domains: one domain is DNaseI-like and the other is a DNA-binding domain comprising two HhH (helix-hairpin-helix) motifs. The following DNA-binding (HhH)_2_ (comprising two tandem HhH motifs) domains were fused to the C-terminus of bovine DNaseI:

1. The C-terminal domain of DNaseTA, hereafter referred to as DT, from an extremely halo-tolerant bacterium *Thioalkalivibrio sp. K90mix*. This fusion hereinafter is abbreviated as DNaseDT.
2. The C-terminal domain of a relatively well characterized competence protein ComEA, hereafter referred to as BS, from *Bacillus subtilis* [7]. This fusion hereinafter is abbreviated as DNaseBS.

Both domains enhanced salt tolerance of DNaseI, albeit to different extent: DNaseTA retained some activity at high salt concentrations (up to 4M NaCl), while DNaseBS was active only at low salt concentrations. Molecular modeling shed some light on this difference indicating that upon domain DT binding to DNA more sodium ions might be transferred from the surface of DNA to solvent compared to the corresponding binding of domain BS.

In this article we present an example where principles of Nature’s evolutionary engineering were applied to enhance properties of a protein that is widely used in biotechnology.

## Materials and Methods

### Phylogeny analysis

#### Selection of sequences

The goal of the analysis was to track the origin of the (HhH)_2_ domains that are detected in bacterial DNaseI-like proteins [6]. Initially, a set of sequences of bacterial proteins that harbor a (HhH)_2_ domain (H3TH_StructSpec-5′-nucleases superfamily, CDD v3.12 [8]) and an exonuclease/endonuclease/phosphatase domain (EEP superfamily, CDD v3.12 [8]) were collected. The sequence fragments that correspond to a (HhH)_2_ domain were used to construct a hidden Markov model (HMM) with HMMER v3.1b1 package [9] and a search was run against UniProtKB [10] via HMM webserver [11]. The bacterial sequences in UniProtKB [10] that matched the HMM with e-value lower than 10^−13^ were clustered at 70% identity using CD-HIT [12, 13]. The clustering cut off and selection stringency were manually adjusted to keep up to ∼100 of final clusters. A protein from Chinese hamster *Cricetulus griseus* (UniProt AC G3HTJ3) was added to the analysis as an out-group. This eukaryotic protein harbors DNaseI-like domain and two tandem (HhH)_2_ domains. The sequence of the domain that best matched the HMM was used for the construction of a phylogenetic tree.

#### Tree building

Only the fragments in the collected sequences that matched the HMM were used to construct the phylogenetic tree. The sequence fragments were aligned using PROMALS3D server [14, 15]. The tree was build using RAxML 8.1.22 program [16]. Automatic protein model selection using maximum likelihood (ML) score based criterion was used. At first, a Bootstrap search using extended majority-rule consensus tree criterion was conducted. After the Bootstrap analysis a search for the best-scoring ML tree was done. This tree was used for further analysis.

#### Tree annotation

CDD v3.12 [8] (server accessed in December, 2014) was used to detect superfamily and multidomain hits in the full length sequences, which regions matching (HhH)_2_ domain were used to construct the phylogenetic tree. ETE 2.2 package was used for the visualization of the tree, the detected domain search hits and supplementary information [17]. The root was placed according to the out-group mammalian (*Cricetulus griseus*) protein.

### Cloning, expression and purification of proteins

Coding DNA of mature, without signal peptide, wild type bovine DNaseI (NCBI reference sequence NM_174534.2) was cloned into a pLATE51 vector using aLICator™ LIC Cloning and Expression system (Thermo Fisher Scientific, #K1271). Primer sequences for one required PCR step are indicated in Table 1. The sequence of the N-terminal His_6_-tag and associated linker was added during cloning and originated from the vector.

**Table 1:** Primers that were used to clone bovine DNaseI and to construct the DNaseI fusions using two-step megaprimer PCR.

Enzyme	PCR step	Primer sequence
DNaseDT	1	CGGTGGAGGTGACGCTGACAGGTAACACGCTGCACGACGC
	1	GGAGATGGGAAGTCATTACTCGATGCAGGCCTCACCAC
	2	GGTGATGATGATGACAAGCTGAAGATAGCAGCCTTCAACATCCG
DNaseBS	1	CGGTGGAGGTGACGCTGACAGGAGAGGAAACAGCAGTGCAGC
	1	GGAGATGGGAAGTCATTACTTTACTGTAATGGAAGACTTTATTTTCTCA
	2	GGTGATGATGATGACAAGCTGAAGATAGCAGCCTTCAACATCCG
DNaseI	1	GGAGATGGGAAGTCCTTATGTCAGCGTCACCTCCACC
	1	GGTGATGATGATGACAAGCTGAAGATAGCAGCCTTCAAC

**Fig 1:**
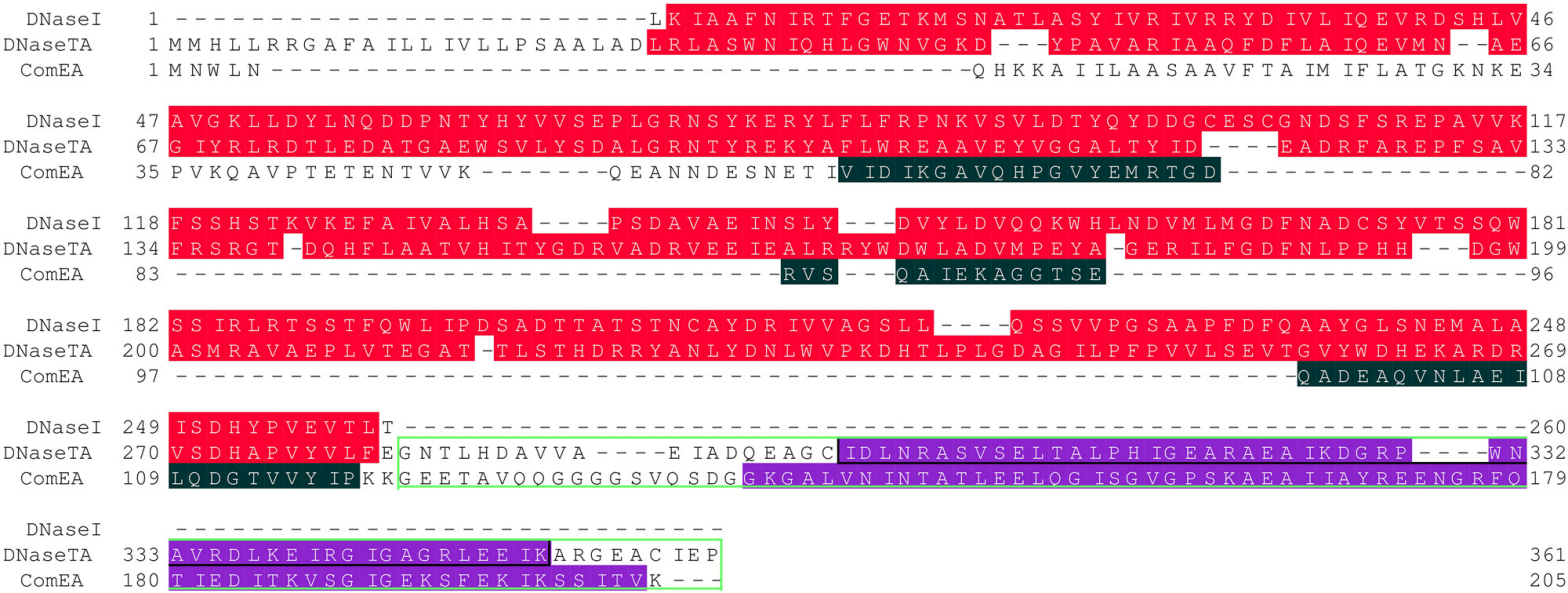
Rationale for sequences of fusions. Sequences in the alignment are: DNaseI—bovine DNaseI (excluding N-terminal signal sequence), DNaseTA—DNase from *Thioalkalivibrio sp. K90mix*, ComEA—competence protein ComEA from *Bacillus subtilis*. Background colours indicate CDD v3.12 hits [8]: red colour indicates region of exonuclease/endonuclease/phosphatase superfamily domain, black colour indicates region of soluble ligand-binding beta-grasp superfamily domain, violet colour indicates region of H3TH_StructSpec-5′-nucleases superfamily domain (two tandem HhH motifs). Green framework indicates sequence fragments from DNaseTA and ComEA proteins that were “attached” to the C-terminus of bovine DNaseI.

Coding sequence fragments of DNaseDT and DNaseBS for cloning were generated by two-step megaprimer PCR. All PCR reactions were performed with 2x Phusion™ Flash High-Fidelity PCR Master Mix (Thermo Fisher Scientific, #F-548S). The PCR products were cloned into a pLATE51 vector using aLICator™ LIC Cloning and Expression system (Thermo Fisher Scientific, #K1271). DNase from *Thioalkalivibrio sp. K90mix* cloned to a vector pLATE31 [6] was used as a template for the first PCR reaction when DNaseDT was constructed, while *Bacillus subtilis subsp. subtilis str. 168* (ATCC 23857) genomic DNA was used in the corresponding reaction for DNaseBS. The resulting fragments were gel-purified and together with the third primer were used in the second PCR reactions where bovine DNaseI cloned to a vector pLATE51 was used as a template. The resulting fragments were gel-purified and cloned into a pLATE51 vector. The sequences of the N-terminal His_6_-tag and associated linker were added during cloning and originated from the vector. Sequences of primers used are indicated in Table 1. The rationale for the resulting protein sequences is given in Fig 1.

Protein expression and purification were done as described in [6]. The constructs were cloned using *E.coli* ER2267 strain (New England Biolabs). For the protein expression the constructed recombinant plasmids were transformed into *E.coli* ER2566 strain (New England Biolabs). For the protein expression the constructed recombinant plasmids were transformed into *E.coli* ER2566 strain (New England Biolabs). LB agar and LB broth used were supplemented with glucose and carbenicillin. For protein expression precultures were grown till OD_600_ 0.3 and then used for inoculation. The expression was induced by addition of IPTG (1 mM) when OD_600_ reached 0.8–0.9 and main cultures were subsequently grown at 23°C for 16 h. Bacteria were lysed chemically and expressed proteins were purified in one step using nickel affinity spin columns (Thermo Fisher Scientific, #88225).

### Characterization of protein samples

#### Purity assay

Concentrations of the target proteins and contaminant proteins in the partially purified samples (His_6_-tag based one-step purification) were determined based on SDS-PAGE (12% gels) and subsequent densitometry. SDS-PAGE was done using hand-casted 15 wells mini gels (Biorad, #165–8011) and Mini-PROTEAN™Electrophoresis System (Biorad, #165–8000).

As a standard for quantification a 250 μg/ml BSA sample was used (Thermo Fisher Scientific, #23208). Two-fold dilution series of BSA from 250 μg/ml to 7.8125 μg/ml was prepared for calibration and corresponding six samples were loaded on gels together with assayed protein samples. Samples were prepared using 4x loading dye (0.25 M Tris-HCl, 1.6 mM EDTA (pH 8.5), 8% (w/v) SDS, 40% (w/v) glycerol, 0.04% (w/v) bromophenol blue, 0.024% (w/v) pyronin Y) and DTT (100 mM final concentration). Before loading samples were heated 10 min at 95°C. Per well 5 μl of samples were loaded. PageBlue™ Protein Staining Solution (Thermo Fisher Scientific, #24620) was used for staining. Before staining gels were fixed for 15 min in solution containing 25% isopropanol and 10% acetic acid.

The assayed protein samples were diluted 10 times (for target protein quantification) and 2.5 times (for quantification of contaminants). Dilutions were done with storage buffer (10 mM Tris-HCl, pH 7.5, 1 mM CaCl_2_, 50% glycerol). Duplicate dilutions were performed for each sample. SDS-PAGE for assayed protein samples along with BSA standards was run twice. Densitometry was done using TotalLabQuant v12.3 software. Background was removed using “Rolling Ball” method with radius parameter set to 400, bands were detected automatically using default parameters and additional bands were added after visual inspection of profiles. “Advanced Gaussian fit” option for profile deconvolution was used.

#### Kunitz assay

Based on SDS-PAGE quantitation protein samples were diluted with storage buffer (10 mM Tris-HCl, pH 7.5, 10 mM CaCl_2_, 50% glycerol) to get 1 pmol/ l concentration. Standardized liophylised DNaseI from bovine pancreas (Sigma, #D4527) were dissolved in the storage buffer to get 0.375 U_k_/ l (U_k_—Kunitz unit).

Reaction was performed following standard Kunitz assay conditions [18]: 83 mM sodium acetate, pH 5.0, 4 mM magnesium acetate, 25 μg/ml *λ* phage DNA. Whole Reaction volume was 400 μl. 20 μl of diluted protein samples or DNaseI standard was added immediately before measurements. Reaction mixes were prepared and measures were taken at ambient temperature (∼23°C). UV absorbance at 260 nm was observed using Evolution™ 220 UV-Visible Spectrophotometers (Thermo Fisher Scientific, #840–210500) for 120 s, measurements were taken each 5 s. Data was fitted into first order equation. Reactions for each sample were performed in triplicates and an average UV absorbance increase rate was calculated. Nuclease activity is proportional to the UV absorbance increase rate; therefore, based on the rates of the reactions with the standardized DNaseI corresponding activities for the assayed protein samples were calculated.

### Activity assays

Activity assays using non-fluorescent DNA substrates were performed as described in [6].

#### Digestion of long unlabeled DNA substrate

2 μg pUC19 DNA cleaved with SmaI was digested with 2.5 nM of relevant DNase enzyme (DNaseI, DNaseBS or DNaseDT). Two ranges of NaCl concentration were explored: 0–1 M in 0.1 M increments and 0–4 M in 0.4 M increments. The reactions were performed for 10 min at 37°C in 100 μl of the reaction mixture with 10 mM Tris-HCl, pH 7.5, 1 mM CaCl_2_, 2.5 mM MgCl_2_. Reaction products were analysed by 1% agarose gel electrophoresis in 1x TAE buffer with ethidium bromide. ZipRuler™ Express DNA Ladder 2 from ZipRuler™ Express DNA Ladder Set, ready-to-use (Thermo Fisher Scientific, #SM1373) was used as a molecular weight standard to evaluate the degradation of the DNA substrate.

#### Digestion of short radioactively labeled DNA substrate

10 nM 16 bp DNA (2 nM were labeled with ^33^P at 5′-end) was digested with 0.66 nM of relevant DNase enzyme (DNaseI, DNaseBS or DNaseDT) at 37°C in 100 μl of a reaction buffer: 10 mM Tris-HCl, pH 7.5, 1 mM CaCl_2_, 2.5 mM MgCl_2_. 9 μl of the reaction mixtures were removed at 1, 2, 4, 8, 16, 32, 64, 128, 192 minutes after start. The samples were mixed with 9 μl of 2x RNA loading dye (Thermo Fisher Scientific, #SM1373), heated for 5 min at 95°C and analysed by denaturing PAGE. The half-life of the substrate digestion was estimated during subsequent densitometry.

#### Digestion of fluorescently labeled short DNA substrate

Activity of DNaseI and its fusion variants at relatively low salt concentrations was evaluated by analysing digestion of fluorescently labelled DNA duplex (30 bp). As a substrate a dual labelled duplex was used that was produced by hybridizing the following single stranded oligonucleotides: 5′-GTTGGTGGGTTTGGGTGTGGGTTTGTGTTT-BHQ1-3′ and 5′-FAM-AAACACAAACCCACACCCAAACCCACCAAC-3′. Reactions were prepared in the following buffer: 10 mM Tris—HCl, 3 mM EDTA, 1% Triton X-100, 1 mg/ml BSA. 0.2 μM of DNA duplex and 0.044 nM concentrations of relevant DNase enzyme (DNaseI, DNaseBS or DNaseDT) were used. Reactions were started by addition of 10X start solution containing 40 mM CaCl_2_ and 100 mM Mg acetate. Before start of fluorescence monitoring 25 μl start solution was added to 225 μl of reaction mix with enzyme. The fluorescence was monitored in 12 seconds intervals for 10 minutes, data (subsets of consecutive 2–4 data points) was fitted into first order equation and a maximum fluorescence change rate was calculated (Gen5™ Reader Control and Data Analysis Software), which was proportional to enzymatic activity. Fluorescence was monitored and start solution was distributed across reaction mixes by Synergy 2 Multi-Mode Reader (BioTek). Reaction mixes were prepared and fluorescence measures were taken at ambient temperature (∼23°C). Such experiments were performed by varying amounts of NaCl in the final reaction buffer (0, 50, 100 mM) in order to estimate activity changes due to increase in ionic strength.

### Testing feasibility for practical applications

We tested feasibility of the two fusions for two practical applications: i) elimination of contaminant DNA during RNA purification, ii) digestion of template DNA after in vitro transcription. DNA digestion effectiveness of the two fusions was compared to commercially available bovine DNaseI (Thermo Fisher Scientific, #EN0525 and #EN0523). Before treatment with nuclease some samples were diluted using the following buffer: 50 mM Tris—HCl, pH 7.5; 0.5% Triton X—100; 0.5 mg/ml BSA; 50 mM CaCl_2_. We determined the amount of DNA in samples by quantitative PCR (qPCR). qPCR was performed using Maxima™ SYBR Green/ROX qPCR Master Mix (Thermo Fisher Scientific, #K0221) with two-step cycling protocol. The primer sequences are indicated in Table 2. The reaction volume was 25 μl (2 μl of samples). 0.3 μM primer concentrations were used. Samples for standard curves were prepared by diluting either human genomic DNA (Thermo Fisher Scientific, # 4312660) or control template DNA for *in vitro* transcription with TE buffer.

**Table 2:** Primer sequences that were used for experiments to test feasibility for practical applications.

Application	Sequence
Elimination of contaminant DNA during RNA purification	GCCACGTCTCCACACATCAG TGGTGCATTTTCGGTTGTTG
Template DNA removal after in vitro transcription	TGATGACGGTGAAAACCTCTGA CGGCATCCGCTTACAGACA

#### Template DNA removal after in vitro transcription

*In vitro* transcription was performed using TranscriptAid™ T7 High Yield Transcription Kit (Thermo Fisher Scientific, #K0441). As a template the control DNA (included in the kit) was used (1 μg per one reaction). After *in vitro* transcription template DNA was digested either adding varying amounts of nuclease preparation (1.3 pmol/ l) directly to the *in vitro* transcription mix (20 μl) or to the same volume of 5 times diluted transcription mix. After addition of nucleases the reactions were incubated for 15 min. at 37°C. The nucleases were inactivated by addition of 2 μl 0.5 M EDTA and heating for 10 min at 65°C. The remaining DNA was determined by subsequent qPCR.

#### Elimination of contaminant DNA during RNA purification

RNA was purified from three arbitrary human blood samples using GeneJET™ Whole Blood RNA Purification Mini Kit (Thermo Fisher Scientific, #K0761). DNA hydrolysis was done directly on the column filter during an additional washing step. In this step varying amounts of nuclease were added together with 100 μl of the following buffer: 22.5 mM Tris-HCl, pH 7.5; 1.125 M NaCl; 10 mM MnCl_2_. The eluted RNA samples (undiluted, 10 and 100 times diluted) were futher analysed by RT-qPCR (reverse transcription and quantitative PCR) detecting RNA and undigested genomic DNA (RT step without reverse transcriptase). Reverse transcription was done using Maxima™ First Strand cDNA Synthesis Kit for RT-qPCR (Thermo Fisher Scientific, #K1671). Then DNA amount after RT step was determined by qPCR.

### Molecular modeling

#### Homology modeling

Spatial structures of BS (from *Bacillus subtilis*) and DT (from *Thioalkalivibrio sp. K90mix*) were modeled using I-Tasser 3.0 [19] and the best models (in terms of C-score) where used for further analysis. The two sequences marked by green frame in Fig 1 were the targets. The low quality N-terminal unstructured fragments (inter domain regions) were discarded from the models based on expected accuracy along the sequence (I-Tasser 3.0 output). The N-terminal and C-terminal regions were further trimmed to remove unstructured terminal coils that do not match in structural superimposition of the two domains. Structural superimposition, manual inspection and trimming of the models was done using Pymol [20].

#### Protein-DNA complex modeling

The resulting domain structures were superimposed (using MultiProt 1.6 [21]) with homologous domain from a crystallographic structure PDB ID: 3E0D [22] (one of templates used in homology modeling) and the 38 bp length DNA fragment close to the superimposed domains was “excised” from the PDB structure for further modeling. The geometry of this fragment was optimised using 3D-DART server (accessed in December, 2014) [23]. Then it was docked to the two DNA-binding domains using HADDOCK server (accessed in December, 2014) with “Refinement” interface [24]. The resulting complexes were used for further electrostatic calculations.

#### Electrostatic calculations

The two modeled DNA-protein complexes were superimposed using Pymol [20] and used for electrostatic calculations using APBS tools 1.4.0 [25]. Results were visualized in Pymol [20]. The structures were prepared for electrostatics calculations by adding hydrogens with PDB2PQR 2.0.0 [26] using Amber force field [27]. APBS tools were used to predict changes in polar solvation term of free energy that occur when protein-DNA complex is formed. The grid parameters for the calculations were same for both complexes. Nonlinear Poisson-Boltzmann equation was used, all other parameters were default. The calculations were done in accordance with the published analogous calculations on RNA-protein interactions [28]. APBS output files (density maps) that correspond to 0.4 NaCl were used to infer changes in local ion and charge concentrations upon formation of complexes between DNA and the two DNA-binding domains. This was done using auxiliary tools from the APBS package.

## Results

### Origin of DNaseTA C-terminal domain

**Fig 2:**
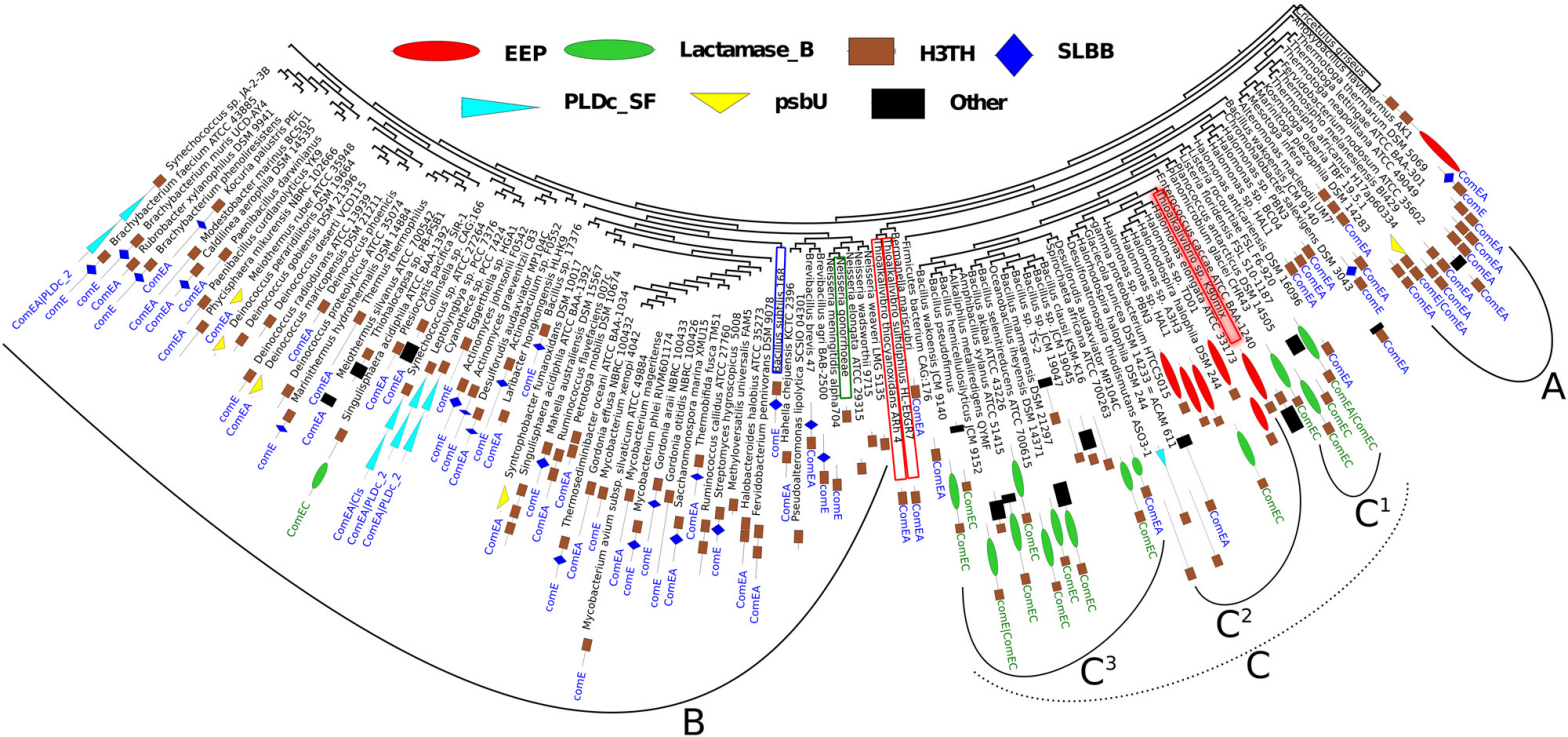
Phylogeny analysis of bacterial proteins that harbor domains, similar to (HhH)_2_ domains (H3TH domains) found in bacterial proteins that comprise one H3TH domain (H3TH_StructSpec-5′-nucleases superfamily, CDD v3.12 [8]) and one exonuclease/endonuclease/phosphatase domain (EEP superfamily, CDD v3.12 [8]). An experimentally characterized ComEA protein from *Bacillus subtilis*[7] is marked by blue frame. Other experimentally characterized ComE protein from *Neisseria gonorrhoeae*[29] is marked by green frame. Leaf names indicate species of the corresponding organisms. Leafs of proteins from *Thioalkalivibrio* genus are marked by red frames. The experimentally characterized DNase from *Thioalkalivibrio sp. K90mix* (DNaseTA) is marked by reddish background [6]. The domain architecture of the corresponding sequence is indicated by figures next to the name of the corresponding species. Next to the figures indicating domain architectures corresponding names of multi-domain CDD v3.12 hits [8] are indicated. Domain architectures are indicated by using superfamily names of CDD v3.12 hits [8], only H3TH_StructSpec-5′-nucleases superfamily is abbreviated as H3TH. EEP denotes exonuclease/endonuclease/phosphatase superfamily, Lactamase_B—metallo-beta-lactamase superfamily, SLBB—soluble ligand-binding beta-grasp domain superfamily, PLDc_SF—catalytic domain of phospholipase D superfamily, psbU—photosystem II 12 kDa extrinsic protein. Hits, which occurred only once, were indicated as Other. Notations A, B, C, C^1^, C^2^, C^3^ are used to describe the tree in the main text.

First we tracked the origin of the DNaseTA C-terminal domain, which contains two tandem HhH motifs. The topography of the resulting phylogenetic tree is given in Fig 2. All the proteins included in the analysis harbor a two tandem HhH motifs comprising domain, which belongs to a superfamily of H3TH domains of structure-specific 5′ nucleases [8] and hereinafter is referenced as a H3TH domain. At the base of the tree there is a group of proteins that harbor multiple H3TH domains from *Thermotogae* phylum, which match multi-domain hits ComEA [8]. This group is denoted as “A” in Fig 2. All other proteins emerge from the sister group. This group further splits into two distinct sister groups (“B” and “C” in Fig 2). The “B” group (Fig 2) contains two experimentally characterized competence proteins: i) ComE from *Neisseria gonorhoeae*[29], which comprises one H3TH domain; and ii) ComEA from *Bacillus subtilis*[7], which comprises one SLBB (soluble ligand-binding beta-grasp domain superfamily) domain and one H3TH domain. In addition to proteins that have such domain organization, the “B” group contains plethora of experimentally not characterized proteins, which harbor other domains in addition to a H3TH domain. ComE-like proteins from *Thioalkalivibrio* and *Halomonas* genera that comprise single H3TH domain are localized at the base of the “C” group (Fig 2). Analysing proteins that are close to the lineage that leads to DNaseTA, we find proteins from *Bacillales* order that have a H3TH domain fused to a metallo-*β*-lactamase superfamily domain (C^1^ in Fig 2). Hereinafter such proteins are refereed as lactamase-*β*-H3TH proteins. Further in the lineage leading to DNaseTA we find two sister groups: i) one (C^3^ in Fig 2) contains lactamase-*β*-H3TH proteins from *Bacillaceae* family; ii) the other one (C^2^ in Fig 2) contains DNaseTA and other proteins that harbor EEP (exonuclease/endonuclease/phosphatase superfamily) domains fused to H3TH domains. In this group (C^2^ in Fig 2) apart from nucleases harboring a H3TH domain (including DNaseTA) we find one lactamase-*β*-H3TH protein. Thus, we have shown that the H3TH domain of DNaseTA originates from proteins that are related to the experimentally characterized bacterial competence proteins and share with them common precursors.

### General characteristics of fusion proteins and their samples

Samples of His_6_-tagged fusion proteins after one step affinity purification were used for further experimentation. Yield of the proteins per mg of wet *E. coli* paste and purities of protein samples are given in Table 3.

**Table 3:** Yield of DNaseI fusions and general characterisation of used protein samples. DNaseDT denotes the fusion with the (HhH)_2_ domain of DNase from *Thioalkalivibrio sp. K90mix*. DNaseBS—the fusion with homologous domain of ComEA protein from *Bacillus subtilis*.

			Specific activity^1^	
Sample	Yield, mg/g	Purity, %	U_k_/mg^2^	U_k_/pmol^2^
DNaseBS	0.24	64	3853	0.158
DNaseDT	0.14	32	4514	0.184
DNaseI	0.35	66	6217	0.201

^1^ based on Kunitz assay [18].

^2^ target protein.

Specific activities of the enzymes are also given in Table 3. Molecular weight of both fusions are higher (41 kDa) than the molecular weight of DNaseI (32 kDa). In order to take this into account specific activities are indicated in Table 3 as Kunitz units (U_k_) per mg of a protein and per pmol of a protein. Specific activities expressed as (U_k_/mg) were lower compared to DNaseI. Specific activities expressed as U_k_/pmol were close, albeit still lower, compared to the activity of DNaseI. It should be noted that Kunitz assay is performed at conditions (5.0 pH, absence of calcium ions, presence of salt) that strongly deviate from optimal reaction conditions for DNaseI.

### Salt resistance of DNaseI fusions

The fusion proteins, DNaseBS and DNaseDT, comprise the sequence of the bovine DNaseI fused to the C-terminal DNA-binding domain of *Bacillus subtillis* and *Thioalkalivibrio sp. K90mix*, respectively Fig 1. We constructed only these two fusions and found them, as given below data indicate, to be functional enzymes.

**Fig 3:**
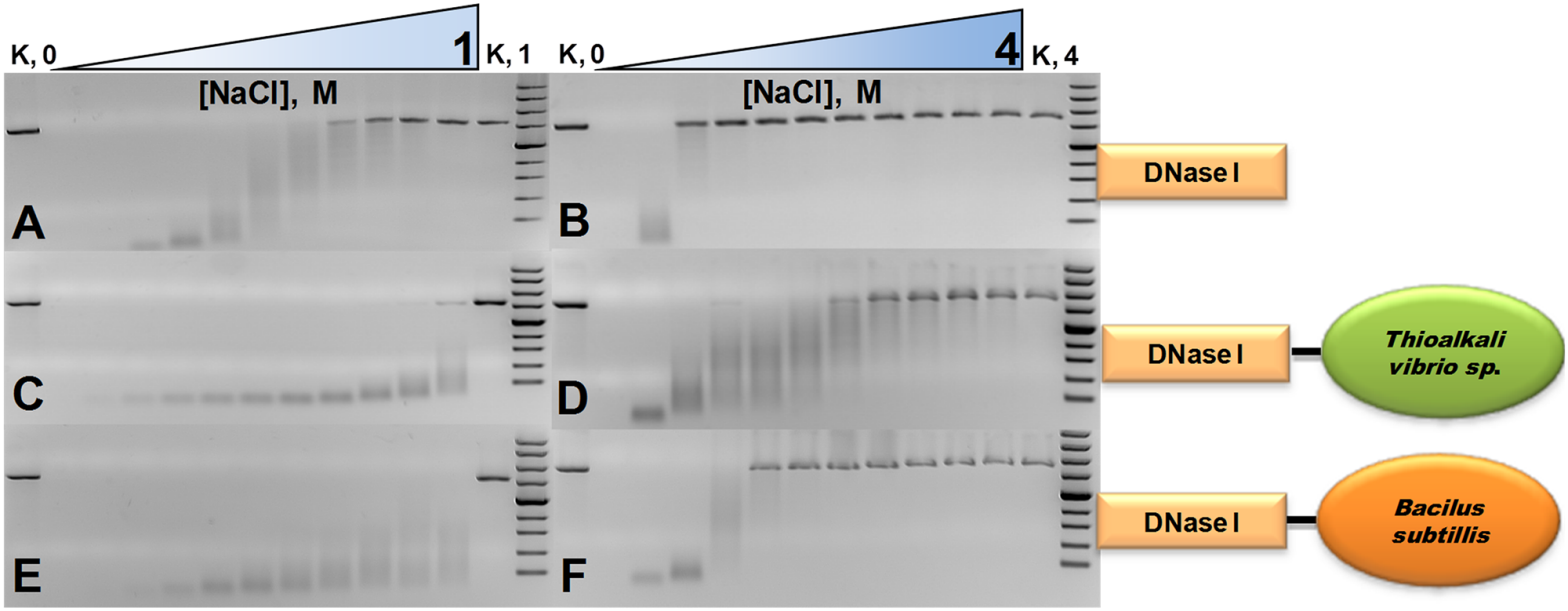
Activity of bovine DNaseI and its two fusion variants at differentionic strengths. Two series of NaCl gradients were used: the first one corresponds to a range from 0 to 1 M with increments of 0.1 M (left electrophoregrams), the second one corresponds to a range from 0 to 4 M with increments of 0.4 M (right electrophoregrams). Activity of analysed proteins was evaluated by digestion of linearised pUC19 plasmid in the presence of various concentrations of NaCl. The data on the fusion with the (HhH)_2_ domain of DNase from *Thioalkalivibrio sp. K90mix* is given at the C and D panels. The data on the fusion with homologous domain of ComEA protein from *Bacillus subtilis* is given at the E, F panels. The data on bovine DNaseI are given at the A, B panels. The electrophoregrams on DNaseI have been already published together with data on DNase from *Thioalkalivibrio sp. K90mix*[6].

**Fig 4:**
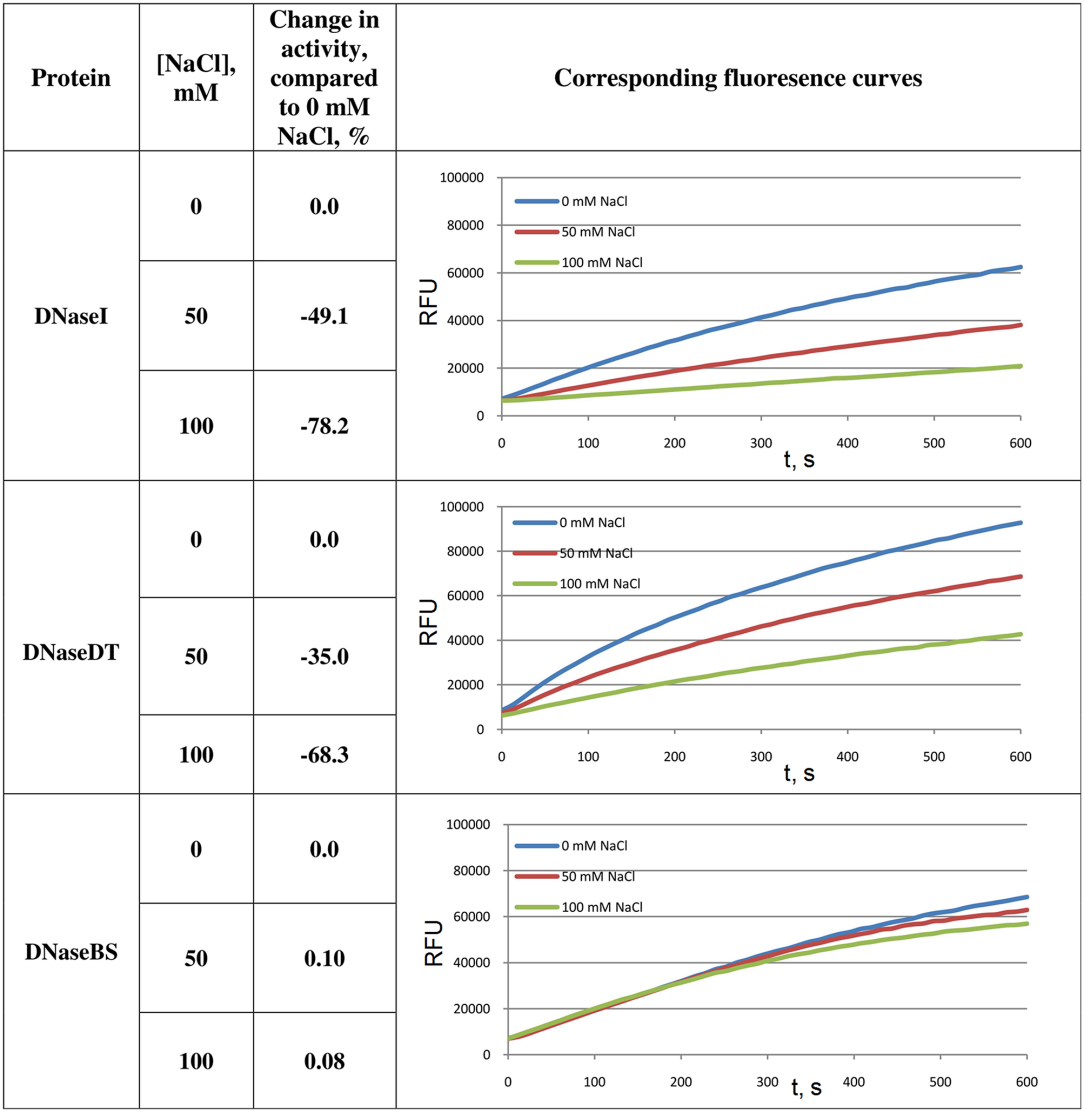
Activity of DNaseI and its fusion with ComEA domain variants at lowsalt concentration. The digestion of labelled DNA duplex (30 bp) was observed. Corresponding activities at 50 and 100 NaCl mM were compared to the activities in the absence of NaCl. Relative fluorescence units (RFU) are plotted versus time. For each curve a maximum fluorescence change rate was calculated, which was considered to be proportional to enzymatic activity. DNaseDT denotes the fusion with the (HhH)_2_ domain of DNase from *Thioalkalivibrio sp. K90mix*. DNaseBS denotes the fusion with homologous domain of ComEA protein from *Bacillus subtilis*.

The activity and salt resistance of the fusions were assessed by digestion of long (Fig 3) and short DNA substrates (Table 4, Fig 4). The quantitative data of radioactive substrate digestion half-life (Table 4) complement the data obtained by electrophoregrams (Fig 3).

**Table 4:** Radioactive substrate digestion half-life of wild type bovine DNaseI and its fusion variants. DNaseDT denotes the fusion with the (HhH)_2_ domain of DNase from *Thioalkalivibriosp. K90mix*. DNaseBS—the fusion with homologous domain of ComEA protein from *Bacillus subtilis*.

NaCl, M	DNaseI	DNaseDT	DNaseBS
	Half-life of 16 bp DNA substrate digestion T_1/2_, min		
0	0.61	0.41	0.70
0.5	732.7^1^	64.41	488.6^1^
1.2	ND^2^	ND^2^	ND^2^
4.0	ND^2^	ND^2^	ND^2^

^1^—Half-life lasts longer than the experiment itself and was computationally inferred from collected data.

^2^—DNA digestion not detectable.

As shown in Fig 3, at 1 M NaCl salt concentration the wild type DNaseI is inactive and does not degrade DNA while both fusion proteins retain activity (the left panels, Fig 3). DNaseDT retains a detectable DNase activity even at 4 M NaCl. However DNaseBS is less tolerant to high salt concentrations than DNaseDT as it retains activity up to ∼1.6 M NaCl (the right panels, Fig 3). The data show that at 0.5 M NaCl DNaseDT is notably more active than DNaseBS and bovine DNaseI. The data in Table 4 also reveal that DNaseDT is more active than DNaseBS and DNaseI at 0 and at 0.5 M NaCl while there is no detectable digestion of the radioactive DNA duplex at 1.2 and 4.0 M NaCl.

The data shown in Fig 4 elucidate differences in activities of the three analysed proteins at relatively low salt concentrations (50 and 100 mM NaCl). In this figure fluorescence curves and corresponding changes in the activities in response to the increased ionic strength are presented. At 0 mM NaCl in the case of DNaseDT the maximum fluoresence increase rate was ∼200% higher compared to the wild type while in the case of DNaseBS it was 95% compared to the wild type. Therefore this data show that at 0 mM NaCl DNaseDT is ∼ two times more active than the wild type enzyme, while the activity of DNaseBS is close to the wild type. However, the data in Fig 4 indicate that up to 100 mM NaCl the digestion of the DNA duplex by DNaseBS is less suppressed by salinity than DNaseDT and DNaseI.

**Fig 5:**
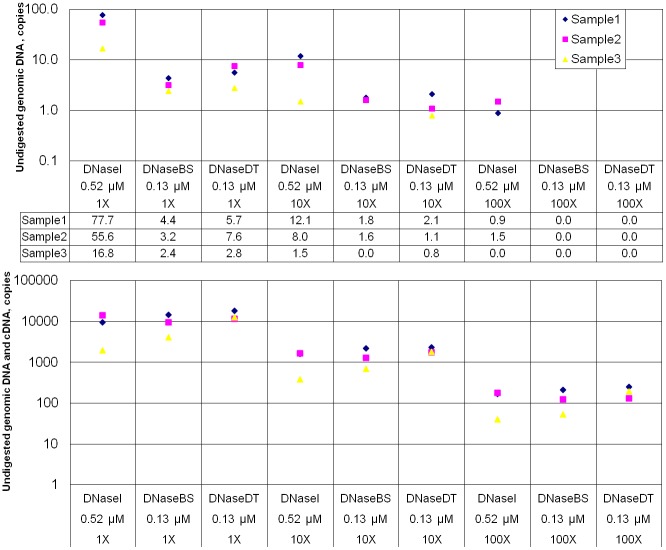
Efficiency of DNA removal by DNaseI and its fusion variants during RNA purification procedure. DNaseDT denotes the fusion with the (HhH)_2_ domain of DNase from an extremely salt tolerant bacterium *Thioalkalivibrio sp. K90mix*, DNaseBS denotes the fusion with homologous domain of ComEA protein from *Bacillus subtilis*. The upper picture represents quantitative evaluation of undigested DNA remaining in eluates (RT-qPCR without added reverse transcriptase). The lower picture represents RT-qPCR results obtained using the same eluates. In the both pictures labels on the horizontal axis indicate the used nuclease, amount of it and the dilution ratio of the eluates before reverse transcription step and quantitation of DNA. A modified protocol of GeneJET™ Whole Blood RNA Purification Mini Kit (Thermo Fisher Scientific, #K0761) was followed and RNA purification columns supplied by the manufacturer were used. During the experiment we have purified total blood RNA. Three arbitrary blood samples were analysed. DNA digestion was performed directly on a filter of a RNA purification column, were respective DNase enzyme was loaded.

**Fig 6:**
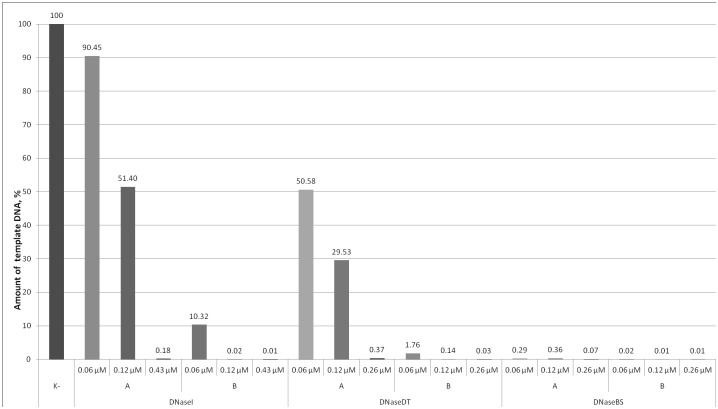
Efficiency of template DNA removal after in vitro transcription reaction using DNaseI and its fusion variants. DNaseDT denotes the fusion with the (HhH)_2_ domain of DNase from an extremely salt tolerant bacterium *Thioalkalivibrio sp. K90mix*. DNaseBS denotes the fusion with homologous domain of ComEA protein from *Bacillus subtilis*. Transcription reactions were performed using TranscriptAid™ T7 High Yield Transcription Kit (Thermo Fisher Scientific, #K0441). As a template 1 μg control DNA from the kit was used. After transcription reaction (2 h, 37°C) undiluted and 5x diluted samples were treated with varying amounts of nucleases. K- denotes a control sample, which was not treated with DNase. A—denotes cases, when sample was not diluted before treatment with DNase. B—denotes cases, when sample was diluted 5 folds before treatment with DNase.

The performance of the fusions was tested in two practical applications: i) digestion of DNA on a membrane during RNA purification (Fig 5), ii) removal of DNA template after *in vitro* transcription (Fig 6). The data reveal that in these experiments the fusion proteins more efficiently removed contaminating DNA than DNaseI. Also we noted that DNaseBS tends to outperforms DNaseDT and less DNA remains undigested. The difference between the fusion proteins is not obvious in the RNA purification experiment (Fig 5), however, the difference is apparent in the DNA removal after *in vitro* transcription experiment (Fig 6).

Thus, the experimental data indicate that at relatively low salt concentrations DNaseBS is more resistant to the increased ionic strength compared to DNaseDT, while at higher salinity the situation is *vice versa*. We employed molecular modeling approach to further elucidate these differences.

### Molecular modeling of the DNA-binding domains

**Fig 7:**
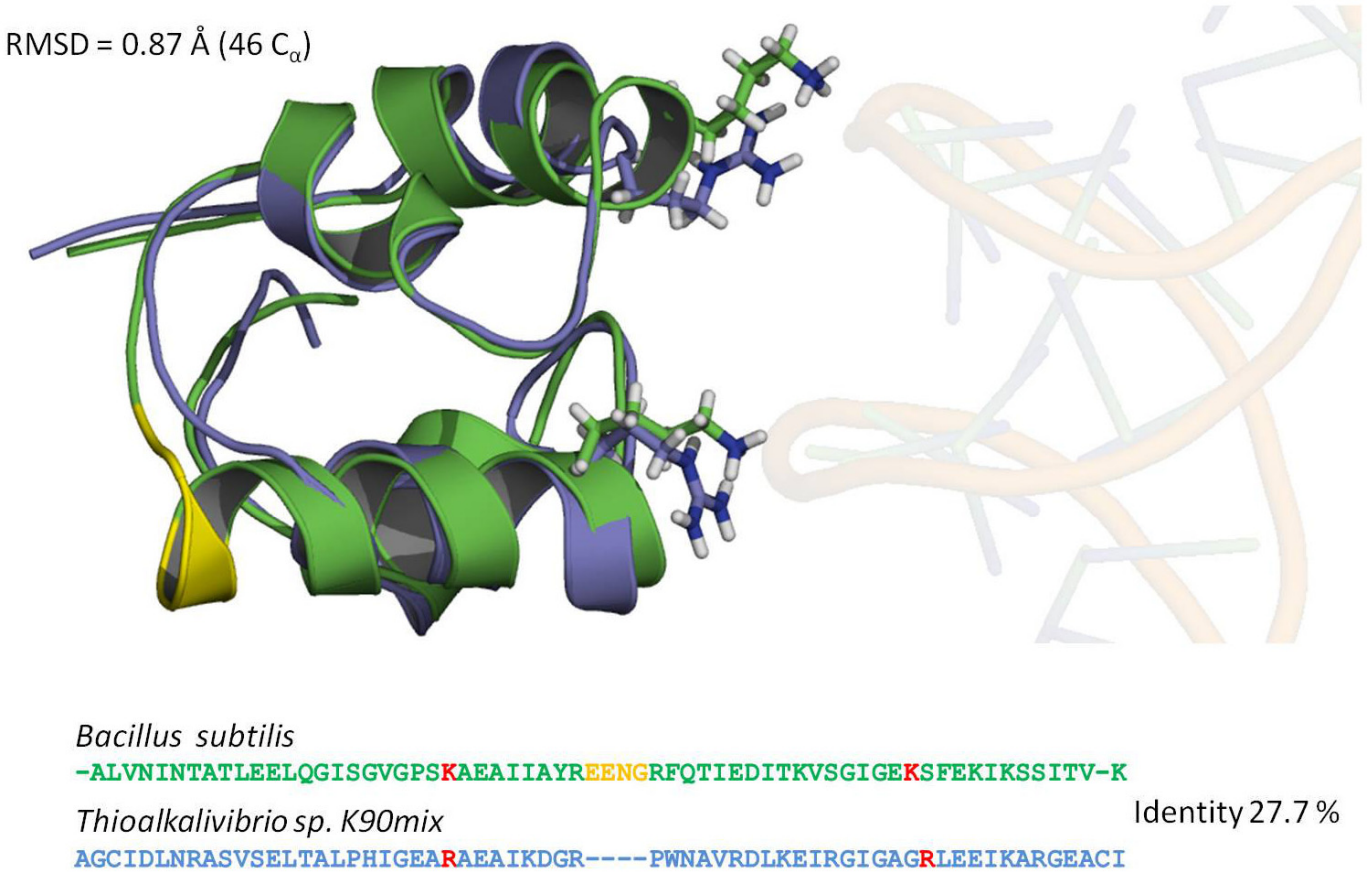
Superposition of structural models of two (HhH)_2_ domains from *Thioalkalivibrio sp. K90mix* (blue colour) and *Bacillus subtilis* (green colour) and corresponding sequence alignment. DNA phosphate contacting positive residues are indicated by red colour in the sequence alignment and by stick representations in the structural alignment. Yellow colour indicates part of the domain from *Bacillus subtilis*, which has no corresponding residues in the domain from *Thioalkalivibrio sp. K90mix*. Approximate position of DNA is indicated by transparent sticks based on structure PDBID: 3E0D after superimposition with the domains.

**Fig 8:**
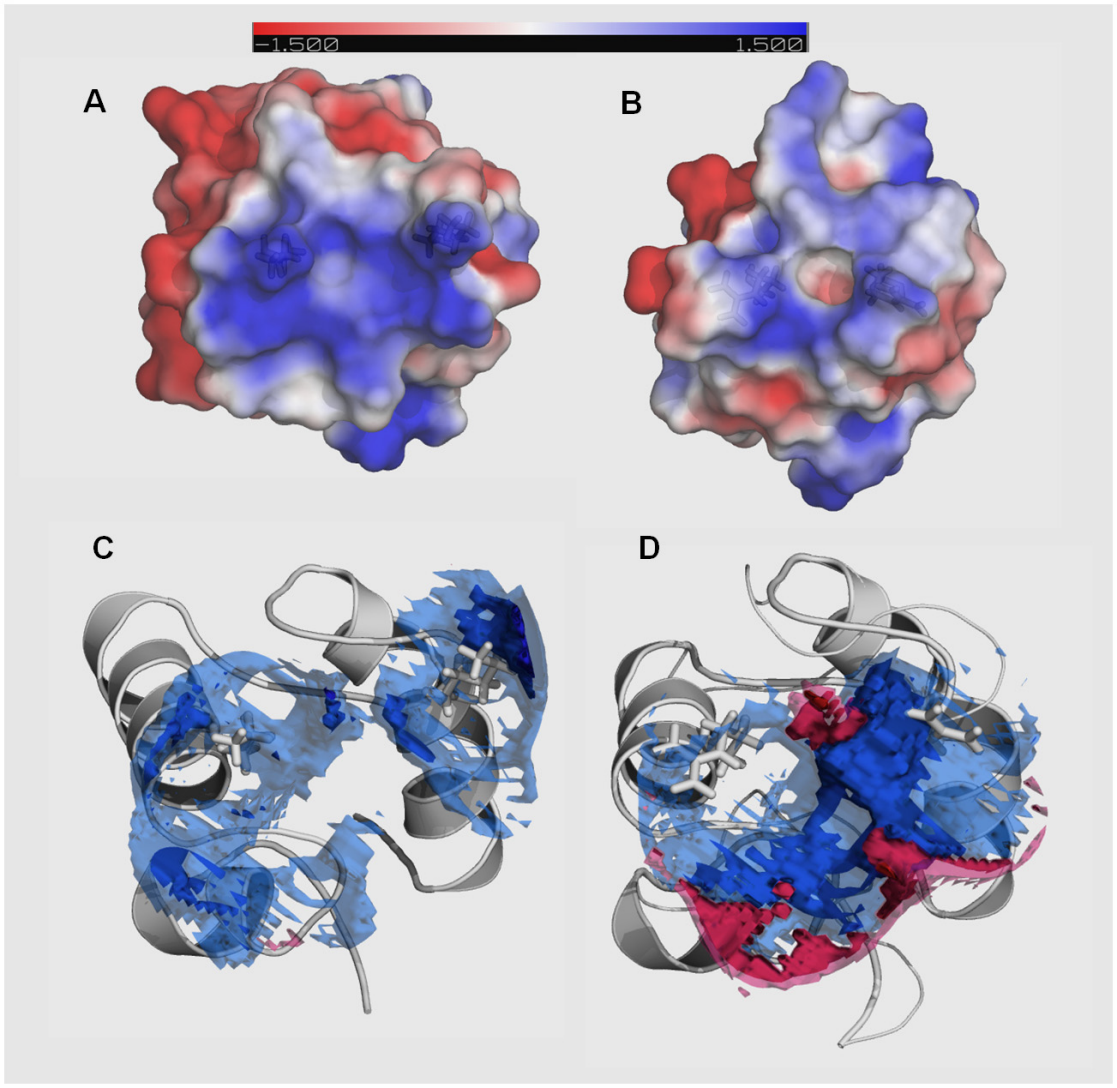
Electrostatic surface potential of DNA-binding surface and changes in local ion concentration upon binding to DNA. Electrostatic potential of DNA-binding surface of the domain from *Bacillus subtilis* is shown on the upper-left (A) and corresponding surface of the domain from *Thioalkalivibrio sp. K90mix* is given on the upper-right (B). The range from −1.5 kT/e in red to +1.5 kT/e in blue was chosen for surface colouring. The surface is semi transparent and stick representations of the DNA phosphates contacting residues are visible: lysines—in case of *Bacillus subtilis* domain and arginines in case of *Thioalkalivibrio sp. K90 mix* domain. The changes in local ion concentrations upon formation of a complex with DNA by the domain from *Bacillus subtilis* are depicted in the lower-left (C), the corresponding changes in the case of the domain from *Thioalkalivibrio sp. K90mix* are depicted in the lower-right (D). The DNA phosphates interacting residues are depicted by white sticks. The isocountour surfaces indicate the changes, which occur in the presence of 0.4 M NaCl. Four isocountour surfaces are visualized simultaneously. The deep blue resembles changes in local ion concentration equivalent to −3 M, the lighter blue resembles changes equivalent to −2 M. Similarly deep red indicates changes equivalent to +3 M and the lighter red indicates changes equivalent to +2 M. Isosurfaces equivalent to +2/-2 M overlaps corresponding isosurfaces which resemble changes equivalent to +3/-3 M.

We have created structural models of the two DNA-binding, (HhH)_2_ domains, i.e. DT and BS, that were fused to bovine DNaseI. The superimposition of the structures along with the corresponding sequences alignments are presented in Fig 7. The sequences are quite different (28% identity), however the modeled structures matches relatively well (RMSD of C_*α*_ is 0.87 Å). However, some apparent differences in the structures are evident. The second HhH motif in BS domain starts with a longer helix compared to the DT domain. The additional residues are marked by yellow-color in Fig 7. In this figure a potential DNA position is indicated. It is evident that both domains have two positive DNA approaching residues: BS has two lysines, DT—two arginines. The matching electrostatic potential of the DNA contacting surfaces are visualized in Fig 8 A (BS) and B (DT) panels. This data indicate that DT from *Thioalkalivibrio sp. K90mix* is more compact and has smaller continuous electropositive patch on the surface than BS from *Bacillus subtilis*.

Further we modeled complexes that can possibly be formed by the two DNA-binding domains and DNA. The resultant models (in PDB format) are given in the supplementary material. This modeling approach revealed that DT can form more hydrogen bonds with DNA than BS (Table 5).

**Table 5:** Hydrogen bonding network between (HhH)_2_ domains and DNA. Two domains were analysed: one from an extremely salt tolerant bacterium *Thioalkalivibrio sp. K90mix*(DT), the other one from *Bacillus subtilis*(BS).

	Protein			DNA			
Domain	Residue number	Residue type	Atom	Residue number	Residue type	Atom	Distance, Å
BS	44	LYS	NZ	31	ADE	O1P	2.89
	73	LYS	NZ	12	CYT	O2P	2.91
	73	LYS	NZ	12	CYT	O5′	2.94
DT	40	ARG	NE	31	ADE	O1P	3.03
	40	ARG	NH2	31	ADE	O1P	3.02
	40	ARG	NH2	31	ADE	O2P	2.88
	66	ARG	NH1	12	CYT	O1P	3.26
	66	ARG	NH1	12	CYT	O2P	2.91
	66	ARG	NH2	12	CYT	O2P	2.91

**Fig 9:**
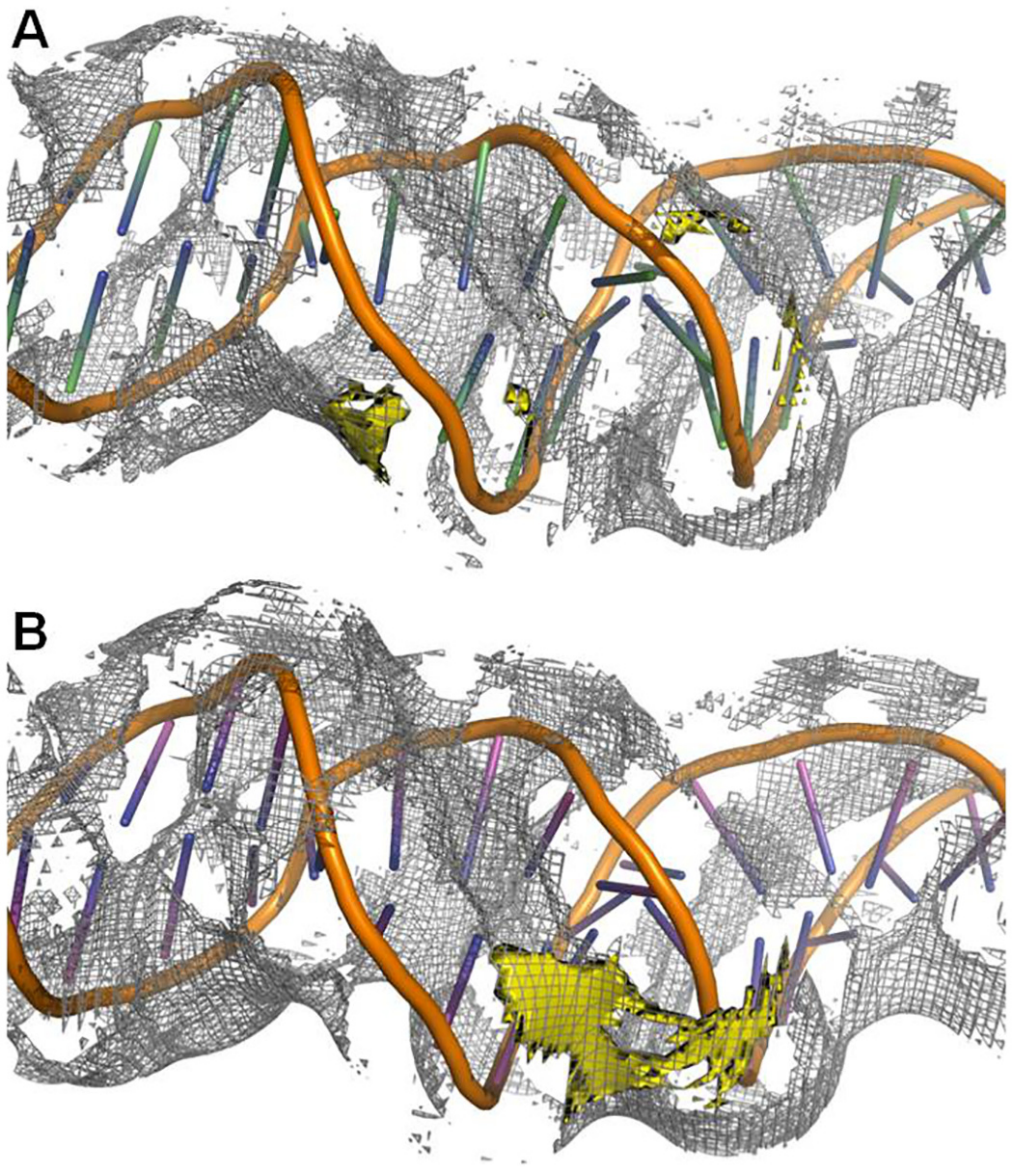
Changes in ion density on the DNA surface upon complex formation. Upper part resembles performed analysis in case of the domain from *Bacillus subtilis* (A), the lower part—in case of the domain from *Thioalkalivibrio sp. K90mix* (B). The white wireframes denotes density of positive charges around the DNA molecule and resembles isosurface at +3 *Me*_*c*_ charge. The yellow isosurface indicates the changes in the ion density upon domain—DNA complex formation. This isosurface indicates area, where local ion concentration decreases by 3 M.

The modeled complexes were further analysed using APBS tools 1.4.0 [25]. Initially we assessed the coulombic contribution to binding. We discovered that in the case of DNaseDT this free energy term is −1093 kJ/mol while in the case of DNaseBS it is about five times smaller (-233 kJ/mol). In order to further elucidate feasibility of DNA-binding we modeled free energy changes that could occur upon the domain binding to DNA (polar solvation term) under several different ionic strength conditions (Table 6). This data indicate that DNA-binding by both domains is not energetically favorable in terms of polar solvation at 0 mM NaCl. At 10 mM NaCl the modeled polar solvation term of the BS (Table 6) is lower than DT, but the values are quite close (−6.79 vs −20.45) at 100 mM NaCl. Further increase in NaCl concentration resulted in much more negative values in case of DT compared to BS. Therefore, at elevated ionic strength DT binding to DNA is much more energetically favourable than BS domain binding (predicted polar solvation term). To further elucidate this phenomenon we analyzed changes in local ion concentrations upon formation of complexes between DNA and the two DNA-binding domains. The results (Fig 8 C, D panels) revealed that upon DT binding to DNA the ion distribution on the protein—DNA-binding surface changed much more than the analogous BS binding. Then we analysed how these changes are reflected in a “cloud” of positive sodium ions that accompany negatively charged DNA (Fig 9). This analysis indicated that upon binding of DT to DNA more sodium ions should be transferred from DNA to solvent compared to the corresponding binding of BS.

**Table 6:** Changes in polar electrostatic solvation energy upon complex formation at different NaCl concentrations. Two domains were modeled: one from an extremely salt tolerant bacterium *Thioalkalivibrio sp. K90mix* (DT), the other one from *Bacillus subtilis*(BS).

	Polar solvation energy changes upon complex formation, kJ/mol		
Concentration of NaCl, M	*BS*	*DT*	Ratio
0	29.10	22.15	0.76
0.01	8.49	13.73	1.62
0.1	-6.79	-20.45	3.01
0.4	-94.71	-20851.04	220.15
0.5	-176.66	-898908.16	5088.42

## Discussion

Recently we have shown that a DNaseI-like nuclease from the extremely halotolerant bacterium *Thioalkalivibrio sp. K90mix* (DNaseTA) retains activity at high salt concentrations and a (HhH)_2_ DNA-binding domain plays the key role here [6]. DNaseTA is a natural fusion of EPE (exonuclease/endonuclease/phosphatase superfamily) and H3TH (two tandem HhH motifs) domains. The primary goal of our study was to construct analogous fusions of bovine DNaseI that would potentially have enhanced halotolerant properties compared to the wild type bovine DNaseI. We were eager to repeat the evolutionary step that created DNaseTA-like nucleases (natural fusion of EPE and H3TH domains). We have chosen DNaseTA as an obvious “donor” of a H3TH domain that could be fused to bovine DNaseI. Additionally, we tracked the origin of the H3TH domain in DNaseTA by searching for other potential fusion partner. The phylogenetic analysis (Fig 2) shows that the H3TH domain originates from proteins that are related to bacterial competence proteins ComE/ComEA, which harbor single H3TH domain or combination of H3TH and SLBB domains, and such domain organization having protein could be a predecessor of the H3TH domain in bacterial DNases, including DNaseTA. The published data revealed two relatively well characterized ComE/ComEA proteins: i) ComEA protein from *Bacillus subtilis*, which is a cell surface DNA receptor (H3TH domain) with the N-terminal fragment (SLBB superfamily domain) anchoring to membrane [7, 8], and ii) ComE protein from *Neisseria gonorhoeae*[29], which comprises single H3TH domain and serves as a part of the machinery that transfers external DNA through periplasm. We considered these proteins as potential “donors” of the H3TH domain for bovine DNaseI and have chosen the ComEA protein from *B. subtilis* due to its domain organization. This protein comprises two domains; therefore, we were able to use the naturally occurring inter-domain linker for our fusion.

Interestingly, the phylogenetic tree shown in Fig 2 indicates that the H3TH domain in the bacterial DNases is closely related to homologous domains in a distinct groups of proteins that have a H3TH domain fused to a metallo-*β*-lactamase superfamily domain (lactamase-*β*-H3TH proteins). A H3TH domain of one lactamase-*β*-H3TH protein is more closely related the homologous domains of the bacterial DNases than to the homologous domains of other lactamase-*β*-H3TH proteins. Thus, it is likely that these proteins inherited the H3TH superfamily domain from the bacterial competence related proteins and then lactamase-*β*-H3TH proteins served as “donors” of the H3TH domain for the nucleases. Ability to catalyse DNA hydrolysis by several metallo-*β*-lactamase domain having proteins was experimentally demonstrated [30–32]. Therefore, it is quite possible that the metallo-*β*-lactamase domain catalyses hydrolysis of nucleic acids, while the accompanying H3TH domain enhances nucleic acid binding properties as it is in the case of DNaseTA. However, this statement requires experimental verification and is out of the scope of this study. The protein from Chinese hamster, which was used in the phylogeny analysis as an outgroup, is another example of potential evolutionary convergence. In this protein two H3TH domains are fused to the EEP domain (DNaseTA has one EEP and one H3TH domain). Thus, evolution has several times combined DNA binding domains with the domains that are similar to DNaseI.

We have selected two H3TH domains to be fused with eukaryotic DNaseI: i) the DNaseTA domain, which has been evolutionary fused with DNaseI-like domain resulting in extremely salt tolerant bacterial DNase [6] (the resultant fusion is abbreviated as DNaseDT); ii) the domain of ComEA protein from *B. subtilis*, which is also a multi-domain protein (the resultant fusion is abbreviated as DNaseBS). In order to create a fusion protein comprising several domains it is critical to select the proper inter-domain linker [33, 34]. In our study we have employed the natural inter-domain linkers of the two multi-domain proteins (Fig 1). This approach proved to be correct since we have tested only one set of the fusions and succeeded in creating functional enzymes.

A DNA-binding domain that is fused to DNaseI might alter DNA binding due to additional DNA binding surface and, indirectly, by interaction between both fused domains. From crystallographic structure of DNaseI-DNA complex [35] it is evident that both N-terminus and C-terminus of DNaseI is in the opposite side from DNA-binding surface. Therefore, the introduced linker region in the created fusions should not physically interfere with DNA binding and, in such a way, reduce activity. However it was showed by Chen et al. [36] that N and C terminuses are crucial for proper folding of DNaseI. Therefore, a DNA binding domain that is fused to the C-terminus could alter folding of the catalytic domain and, subsequently, DNases’s activity. Unfortunately, due to lack of suitable templates, we couldn’t model the inter-domain region and explore the the inter-domain interactions of the created fusions. Therefore the indirect effect of the fused DNA binding domains to DNaseI still remains to be explored.

As indicated in Table 3 yield of both fusions were lower compared to DNaseI. One step purification (based o His_6_-tag) resulted in protein samples with some off-target proteins (purity 32–66%, Table 3). Due to this fact we quantified the target protein using SDS-PAGE and densitometry. Identically expressed and purified samples of a nuclease with an active site mutation didn’t degrade neither long nor short DNA substrates [6]; therefore, we considered that one step purification resulted in functionally pure protein samples and enabled us to analyse properties of the fusions.

We didn’t experimentally evaluate DNA-binding by the created fusions; however, the experiments performed clearly indicate that the DNA-binding domains increase salt tolerance of DNaseI, without reducing activity. Similar salt tolerance effect was induced by adding additional positively charged residues onto DNA-binding surface of human DNaseI that formed favorable interactions with DNA phosphates [1]. Therefore, most likely the enhancements in salt tolerance by the added domains are due to enhanced affinity for DNA of the fusions.

Therefore, both of the fusions were shown to be more salt tolerant than bovine DNaseI, albeit to different extent: the H3TH domain of DNaseTA enabled detectable catalytic activity even at 4M of NaCl, while the fusion harboring the H3TH domain of ComEA protein from *B. subtilis* was evidently less salt tolerant at this concentration. The differences could be partially explained by the origin of the two H3TH domains. The H3TH domain harbored by DNaseBS originates from the ComEA protein of *Bacillus subtilis*. This protein is located on the outer cell surface; thus, it is exposed to the outside environment acting as a DNA receptor [7, 37]. *Bacillus subtilis* can tolerate some fluctuations in salinity, but grows best in low salinity enviroment and is not a high salt tolerant bacterium [38]. The natural environment of this bacterium is soil. In soil the salinity increases episodically in conjunction with desiccation. In contrast, the H3TH domain harbored by DNaseDT originates from an extremely salt tolerant bacterium *Thioalkalivibrio sp. K90mix*. The natural environment of this bacterium is soda lakes—an extremely saline habitat [39]. Thus, it is likely that the salt tolerance of the two fusions DNaseDT and DNaseBS differ because the corresponding H3TH domains were evolutionary adapted to bind DNA at different salinity.

The results of molecular modeling provide some insights on molecular basis of the apparent differences in the salt tolerance of the two fusions. Both H3TH domains bind DNA phosphates with two positive residues, albeit the H3TH domain from *Thioalkalivibrio sp. K90mix* potentially forms more hydrogen bonds (Table 5). The differences became more evident when we modeled changes in polar electrostatic solvation energy upon complex formation at different NaCl concentrations (Table 6)—at 0.5 M NaCl the energy term differed by more than 5000 times (note that it was molecular modeling of one free energy term, not experimental evaluation). The modeling predicts that upon binding of the H3TH domain from *Thioalkalivibrio sp. K90mix* to DNA more sodium ions can be released to the solvent, compared to the corresponding binding of the H3TH domain from *Bacillus subilis* (Fig 9). This nicely agrees with the experimental data that show DNA-binding by two DNA polymerases: more sodium ions were released in case of a more salt tolerant polymerase [40].

In the absence of salt DNaseDT was showed to be more active than DNaseBS by shorter half-life when short radioactively labelled duplex DNA was digested (Table 4) and by faster degradation of fluorescently labeled duplex (Fig 4). We have no clear explanation for this. However, it might be due to more favorable coulombic interactions (∼5 times) between DNA and DT domain compared to BS domain.

Additionally, we still can not clearly understand why DNaseBS is more resistant to increases in ionic strength than DNaseDT at relatively low salt concentrations (up to 100 mM NaCl) (Fig 4). However, we could explain the difference by presuming that the domain from *Bacillus subtilis* efficiently interacts with DNA at low salt concentrations while the homologous domain from *Thioalkalivibrio sp. K90mix* acts in its full potential only at high ionic strength [6].

Proteins tend to accumulate negatively charged surface residues during the adaptation to high salt concentrations [41, 42]. However, in this case the surface electrostatic potential of the H3TH domain from the extremely salt tolerant bacterium (DT) is only slightly less electro-positive than the homologous domain (BS) from the less salt tolerant bacterium (Fig 8)—no extensive acidification is evident. This is in agreement with the analysis done by Becker et al. [43]. In this study acidification of nucleic acid binding pocket was not observed in TATA-binding protein and ribosome elongation factor, when proteins from halophilic and mesophilic organisms were compared.

Apart from the surface acidification, adaptation to high salt environments provokes reduction in the number of surface lysines and their replacement by arginines [44–46]. Researches explain this phenomenon by salt-dependent stabilization [46] or by higher water binding capabilities by arginine [44]. Our study reveals that in case of DNA-binding proteins arginine could be more preferable than lysine due to its ability to form more hydrogen bonds and better shield DNA backbone from counterions.

Our study demonstrates that mimicking domain structure of DNaseTA by fusing bovine DNaseI with DNA-binding domains resulted in the enhanced salt tolerance of the eukaryotic enzyme. The engineered bovine DNaseI was capable to retain some of its activity even at extremely high salt concentrations (4M NaCl). The presented salt tolerance enhancement approach in combination with additional positive residues [1] would potentially boost the salt tolerance of eukaryotic DNaseI to unprecedented levels.
